# Participation of nurses in research development

**DOI:** 10.4102/hsag.v28i0.2360

**Published:** 2023-08-28

**Authors:** Justin O. Rojaye, Robert T. Netangaheni

**Affiliations:** 1 Department of Health Sciences, Faculty of Human Sciences, University of South Africa, Tshwane, South Africa

**Keywords:** clinical, experience, hospital, nurses, participation

## Abstract

**Background:**

Nurses’ experience in participation in research has been very diverse and culturally dependent. A shifting environment that appreciates and supports research growth necessitates studying who is involved in research and how and assessing present individual and organisational research capabilities.

**Aim:**

This study aimed to ascertain the existing research capacity among nurses in a large public hospital in Ijebu Ode, Ogun State, Nigeria, to inform the development of a programme towards building a sustainable research culture.

**Setting:**

A public hospital at Ijebu Ode, Ogun State, Nigeria.

**Methods:**

A qualitative research design was utilised. Data were collected through semi-structured interviews with 21 nurses from the general hospital. The data were then analysed thematically.

**Results:**

Participants highlighted the need for more nurses to be engaged in research development, research development problems and recommended solutions. The critical requirement was that research has a direct impact on clinical practice.

**Conclusion:**

The results from this study show that research development allows nurses to participate in research relevant to their practice and objectives. More focus should be placed on developing and implementing context-specific nursing research agendas and implementation research skills.

**Contribution:**

The overall implications or benefits to the practice (as an example) with reference to the expanded nurses’ clinical knowledge in participating in research and expand nurses’ clinical knowledge in participating in research.

## Introduction

### Background

Over the past decade, there has been a growing drive to guarantee that research results in meaningful, timely benefits for patients, health professionals, healthcare organisations and politicians. The development of research necessitates that much of the research be conducted in the context of practice, where nurses must be involved and take a lead role in research that affects their job and the results of their care (Graham et al. 2018). Over the previous two decades, several assessments of nurses’ engagement in research have been done (Hagan & Walden 2015). The evolving emphasis of these studies recounts a changing culture and expanding activity, with a move from nurse academic research to integration into the role and expectations for clinical nurses (Chen et al. 2019). Lode et al. (2015) conducted a systematic review of research activity and capacity building in clinical nurse practice and identified three critical features: failure to ensure research quality and standards, lack of knowledge about how to increase research capacity, culture and collaboration, and how to increase and organise research utilisation. Building research capacity is a complicated task that requires ongoing multi-level participation. It involves the development of knowledge and expertise, as well as policy, infrastructure and teams, under the robust management of dedicated leaders (Chen et al. 2019).

Despite an increasing appreciation of the importance of research in assuring best practice results, nurses’ participation and effort in research seem to be restricted. More precisely, no study has been conducted to analyse how the rising focus on developmental research has influenced the duties and activities of nurses in general and circumstances in particular. According to Chen et al. (2019), a lack of clarity on essential topics, a lack of relevant instruments and the need to consider context have been barriers to comprehending nurses’ study engagement. In light of these concerns and changes, this study aimed to investigate clinical nurses’ experience towards research, the nature of their involvement in research, and the contextual factors that inhibit or promote participation in research within Ijebu Ode, Ogun State, Nigeria hospital. It will be impossible to build and implement a meaningful research framework and strategies that will optimise nursing research input and outcomes in the future without comprehensive knowledge of the current state of play in terms of who is doing research, where interests and opportunities lie and what level of support is available.

## Aim

The study explored current research capacity and competency and the prerequisites to promote, support and allow a hospital-wide integrated sustainable nursing research culture. In addition, the study sought to address the following research questions. The study explored nurses’ experiences with research, the nature of their involvement and the contextual variables that restrict or support their participation.

## Research methods and design

An interpretative description is a qualitative method that focuses on achieving a specific practice objective using information from all accessible sources (Creswell 2018). This study aims to collect data that will help nurses enhance their research engagement, raise the volume and quality of research they do, and guarantee that research has relevance for clinicians and patients. The qualitative technique was chosen because it detailed descriptions of the participants’ experiences (Brink, Van der Walt & Van Rensburg 2018). One distinguishing element of qualitative research is that it is naturalistic and context-based, focusing on actual situations where interactions occur (Maree 2018). Qualitative research is widely utilised when the study aims to learn people’s opinions on the world. Furthermore, it answers queries regarding people’s experiences, viewpoints and meanings (Creswell 2018). Therefore, the qualitative research approach was the best study paradigm because it enabled the researchers to concentrate on explaining, interpreting and comprehending the elements influencing nurses’ engagement in research.

### Setting

The research was conducted at a hospital in Ijebu Ode, Ogun State, Nigeria. The Ijebu Ode General Hospital is a referral hospital. The hospital is one of the oldest hospitals that manages all medical and surgical patients. The hospital is the largest in the Ijebu area and has about 250 beds.

### Population

The participants comprised nurses who worked full-time at the hospital. The study’s accessible population consisted of 21 nurses who provided healthcare services to patients at Ijebu Ode General Hospital. Participants who satisfied the following criteria were chosen using a purposeful nonprobability sampling technique:

- be a registered nurse who provides healthcare to patients at Ijebu Ode General Hospital
- have worked there for more than a year and
- be willing to participate and sign an informed consent form.

### Data collection and tools

The author employed a variety of strategies to make potential participants aware of their projects, including approaching participants through each unit manager through emails. Semi-structured telephone interviews lasted roughly 50 min each. Data saturation was obtained with the 15th participant after using probing questions. The researcher gathered data between August and September 2022 by obtaining a list of nurses working at the hospital from the Director of Nursing in charge of the institution. The researcher contacted the nurses through a work WhatsApp group and explained the goal and scope of the study. Nurses who had questions or wanted to participate were asked to contact the researcher directly. Before conducting each interview, permission was obtained. The focus group interview lasted 45 min – 50 min, and the interview guide was not changed. Three focus group interviews with seven participants in each group were completed telephonically. Field notes were taken immediately after each interview. With the participants’ agreement, the interviews were transcribed verbatim. The author declared that he is an insider; a researcher is considered an ‘insider’ when he or she shares particular attributes with the participants of the study. A researcher is considered an ‘outsider’ when he or she does not belong to the group to which the participants belong (Braun & Clarke 2019). Although ‘bracketing’ was eliminated by using the ID method (Reiners 2012).

### Data analysis

The data were collected using a semi-interview guide. The raw data from the data were verbatim transcribed before being analysed utilising Braun and Clarke’s (2019) six phases of data analysis:

- familiarisation with the data via reviewing interview transcripts, listening to recordings numerous times, and taking notes from the data; familiarisation with data also include manual transcribing of the recorded interviews (Burdine, Thorne & Sandhu 2021);
- generation of initial codes to define the content and identify and label data aspects important to the research issue;
- searching for themes in the data of all the interview transcripts by reviewing the coded data to identify areas of similarity and overlap;
- reviewing potential themes by comparing them to the organised extracts of the data and determining if the themes work in relation to the data;
- defining and naming themes by summarising them in a few sentences to produce a coherent and overall account of the data. Lode et al. (2015) created a report based on data analysis by creating a clear, persuasive and sophisticated explanation of the data;
- finally, the primary theme and subthemes were determined by the researcher.

### Trustworthiness

The level of trust in the facts, interpretation and procedures employed to assure research quality is called trustworthiness (Polit & Beck 2021). Credibility, transferability, dependability and confirmability were examined to establish the study’s trustworthiness. Member verification was used to establish credibility (Brown 2017). During member verification, the researcher shared the data with the participants to ensure correctness and coherence with their experiences. The researcher also gained credibility by offering a complete account of the procedures and techniques used in the study. Transferability was achieved by adequately defining the study’s context and evaluating the data’s representativeness. Sample characteristics were obtained by choosing a diverse group of participants who provided rich, all-encompassing data to identify saturation levels. As a result, eligible registered nurses were selected to participate in the interviews. Dependability was accomplished by establishing a clear and thorough explanation of the procedures during data collection so that other reviewers could validate the outcomes. The impartiality of research throughout data collection and analysis is referred to as confirmability (Creswell 2018). Confirmability was achieved by accurately and thoroughly documenting the processes, procedures, themes and subthemes.

### Ethical considerations

The College of Human Sciences Research Ethics Review Committee (CREC) of the University of South Africa approved the research (reference number: 60825588_CREC_CHS_2022). This study followed the Code of Ethics for conducting research with human subjects. The hospital authorities and all the participants consented in writing. The participants were also guaranteed anonymity and confidentiality. Therefore, minimal psychological discomfort (risk or harm) may have happened when participants realised that they have missed or had no voice regarding one of their important roles and functions to optimise evidence-based practice. There is some benefit that may occur from being involved as a participant with reference to the significance of the study (Rajan, Jonathon & Peter 2021).

## Results

According to most participants, nursing research significantly impacts the present and future professional nursing practice, making it a vital component of the training process. According to participants, nursing research is essential to the nursing profession and is required for ongoing improvements that support effective nursing care. However, all participants agreed on the low number of nurses engaging in healthcare research development in Nigeria, particularly human immunodeficiency virus (HIV) and acquired immunodeficiency syndrome (AIDS) research. Participants said that nurses do not consider healthcare research and policy formulation a nursing duty; instead, they believe that nurses exclusively care for their patients and that everything beyond the bedside is not their responsibility. Although participants agreed that each nursing function had various duties, the fundamental objective of a professional nurse remains the same to be the client’s champion and deliver the best care based on scientific data (Table 1).

**TABLE 1:** Theme and subthemes.

Theme	Subthemes
Participation in research creation is limited	Few nurses are involved in research development. Challenges of research development. Propose solutions to research creation.

### Theme 1: Participation in research creation is limited

The theme is divided into three categories: few nurses are engaged in research development, the obstacles to healthcare research development, and the invention of research solutions. The subtheme illustrated the degree to which nurses contribute to knowledge development, the accompanying problems and the proposed solutions to involve nurses in knowledge creation actively. There were parallels and contrasts in participants’ impressions of each other, which is examined more below. According to Mundy and Pow (2021), there is a need for strong leadership, an organisational and supporting infrastructure, and research competence development among nurses to assist early career researchers. Research techniques, programmes and cooperation between academic and clinical leaders in this environment seem critical (Mundy & Pow 2021).

#### Subtheme 1: Few nurses are involved in research development

Participants primarily understood the advantages of knowledge generation via healthcare research. Nurses mainly gather and supervise data acquired in their numerous research initiatives. Most of the nurses in this study admitted to having no meaningful involvement in HIV and AIDS research forums. According to participants, nurses use research; we do not create research. However, participants said that a few unit nurse leaders were involved in some healthcare research and management meetings at the establishment. Despite this, none of these nurses were active in HIV and AIDS research. The participants agreed that healthcare research is essential for the profession’s advancement and that meaningful healthcare research participation is vital, alone and in partnership with others. The following are actual quotes that back this up:

‘“I am not one of those who organise these studies; they write the proposals and give them to us at the state, and we assist in monitoring the data acquired.” As a result, other than anything related to my professional role, I am not participating in any independent or private research.’ (Participant 3, 45 years old, female)

‘We can’t develop our profession without research, yet I’m not participating in any HIV research.’ (Participant 2, 42 years old, male)

‘Aside from my knowledge production, I am not participating in research, and it is not concerning HIV.’ (Participant 3, 45 years old, female)

‘I understand the importance of locally created evidence. But unfortunately, we are relying on study data established elsewhere today in practice. We don’t even consider whether the evidence may be applied to our situation most of the time. We would not have such a high illness burden if we could develop our proof by our environment and culture.’ (Participant 1, 40 years old, female)

‘Research was not part of our training program while I was there.’ (Participant 6, 44 years old, female)

‘You know, Mr., I’ve worked with nurses for a long time. But, apart from school projects like yours, I’ve never seen a nurse; I mean, any nurse in any healthcare setting initiate a research study that would pique the authority’s attention and prompt them to declare, “This study is worth sponsoring.”’ (Participant 7, 45 years old, female)

The participants were generally aware of healthcare research’s relevance in addressing population and healthcare system concerns; however, they were not directly engaged in healthcare research investigations. Furthermore, most individuals said their healthcare research involvement ended their school assignments. Nevertheless, the participants revealed that healthcare research played an essential role in the nursing associations’ aims and objectives, which required members to participate in healthcare research with nurses and other health team members, disseminate specialised knowledge nationally and internationally through healthcare research reports and publications, and use healthcare research findings for evidence-based practice and education. On the other hand, participants believed that nurses had a limited role in knowledge development. Although nurses have grown more aware, talented and well-educated in recent decades, they have had little engagement in legislative processes and political choices impacting healthcare delivery. As the bulk of the healthcare staff, nurses strengthen the health system (Hajizadeh et al. 2021). Although nurses’ involvement in health policymaking is evident, few participate in healthcare research processes, especially in the clinical environment. Nurses may use this discovery to create empowering initiatives that will allow them to play more effective roles and improve their engagement in healthcare research development. Furthermore, the retrieved elements in this study may situate nurses in a favourable position and make them potential agents in altering policymaking methods (Hajizadeh et al. 2021).

#### Subtheme 2: Challenges of research development

All participants shared this category. All identified impediments may be divided into two categories: individual and systemic. The term ‘unique hurdles’ refers to certain personal qualities that restrict a nurse’s capacity to participate in policy creation, such as a lack of knowledge or a weak educational background. Systemic impediments appear as organisational structure issues, system politics and the exclusion of certain professions from crucial management roles. Participants noted the shortage of nurses in important research development positions, the lack of invites to research discussions and a nursing directorate at the federal level. Participants responded that those choices were decided for them, even on topics directly impacting nurses, demonstrating a lack of value accorded to nurses’ participation. Participants remarked that the absence of invites to research formulation was common. Participants responded that the obstacles connected with knowledge development are apparent issues, such as a lack of healthcare research expertise and financing. Participants agreed that insufficient school preparation is to blame for poor healthcare research knowledge. Furthermore, practically all participants identified a lack of finance as a hurdle. Surprisingly, none of these nurses had ever submitted a study request for funding to their institution or other organisation. The following are actual quotes that back this up:

‘Nurses encounter several hurdles in producing research and policy, including finance.’ (Participant 4, 43 years old, female)

‘“Nurses don’t want to engage in research because they don’t get paid enough.” That was the sense I received from every nurse I spoke with. And they tell you straight out, “I’m not being paid enough to perform research.”’ (Participant 8, 45 years old, female)

‘“If you’re going to overwork your nurses by incorporating research development into our work, you’d best pay more.” But, on the other hand, if you put nurses through extreme research development stress circumstances, pay them more, which is just reasonable.’ (Participant 9, 40 years old, male)

‘We don’t have the time or resources to engage in healthcare research or policy creation.’ (Participant 5, 42 years old, female)

‘Lack of resources, such as the internet, computers, printers, and access to evidence-based articles to be utilised as a guide in producing research and policy, are significant obstacles for us.’ (Participant 12, 45 years old, female)

‘A lack of motivation on the side of nurses to engage in research since there is no incentive from the government or the institution.’ (Participant 16, 45 years old, female)

Some participants believe that the lack of nurse engagement in healthcare research development is due to a lack of recognition and thanks from management and other healthcare administrators. Nurses often want to deliver the best care possible but might not feel recognised for their hard work and compassionate care attempts. In addition, many interviewees stated that poor earnings were a significant factor in nurses’ lack of research development. Participants suggested different options for appreciating the work of nurses. One way is to introduce rewards and incentives. Others claimed nurses would be appreciated if spoken to in ‘encouraging words’, by ‘offering breaks’ or ‘patting nurses on the back’. In underdeveloped countries, healthcare research is always in progress. However, researchers confront difficulties in selecting a study subject, statement, et cetera.

Furthermore, researchers encounter problems related to expansion, infrastructure limitations and budgetary constraints. As a result, healthcare research and human capital development in Nigeria confront huge hurdles (Campbell & Okuwa 2019). These difficulties include insufficient money, facilities and materials, a lack of knowledge, a lack of application of research findings, a poor ranking in human capital indexes, brain drain and so forth. Therefore, the country’s education policies and programmes must undergo a radical and far-reaching healthcare research overhaul to tackle these problems. Education investment results in a more significant rise in human capital or human resources (Campbell & Okuwa 2019).

According to Hajizadeh et al. (2021), one of the major causes of nurses’ non-participation in healthcare research is a lack of understanding. Hajizadeh et al. (2021) state that poor knowledge and skills in research assessment and insufficient expertise in healthcare research formulation guidelines impede nurses’ engagement in health policymaking (Hajizadeh et al. 2021). The most reported reason in research was a lack of resources. A factor influencing nursing leaders’ engagement in healthcare research was a lack of accessible resources. Furthermore, a lack of support from other sectors, such as the political sector, government officials or professional organisations, impeded nurses’ minimal engagement in policymaking. Most variables influencing nurses’ engagement are connected to management and organisational aspects, as highlighted in the included research (Hajizadeh et al. 2021). The formation of research activities requires a supporting organisational framework. Therefore, inadequate engagement of nurses in policymaking processes will persist in the future and many nations (Hajizadeh et al. 2021). Nurses must recognise the significance of empowerment and involvement in healthcare research development. Creating an environment for nurses to engage with policymakers, decreasing the strain of their tasks and using suitable leadership tactics may all benefit nurses in this area. Furthermore, the identified elements might be used to design instructional programmes to enhance nurses’ knowledge and abilities (Hajizadeh et al. 2021).

#### Subtheme 3: Propose solutions to research creation

Participants said that healthcare research creation solutions for nurses must be explored both inside and outside of nursing. They presented several alternatives that they believed would assist nurses in relieving their workload. They argued that employers and the government should encourage nurses to participate in healthcare research creation to satisfy the demand for HIV and AIDS healthcare in Nigeria. All the participants agree on empowering nurses’ research ability. This category covers participant-identified research empowerment tactics, such as incorporating research into the educational curriculum. However, this approach would only assist individuals starting in the nursing field. Some participants said workshops and in-house seminars should enhance nurses’ research ability because classroom instruction does not give adequate expertise to do research. Some participants advocated for funding designated explicitly for nurses working in research development. Participants develop strategies for successfully including nurses in policy formulation. These initiatives included the creation of a curriculum, incorporating policy courses into higher education programmes, and group campaigning. Some participants, for example, advised beginning with a policy-sensitive curriculum at the training school. Participants largely agreed on the need to develop nursing research capacity via workshops to give funds to urge nurses to act and identify research mentors. Building capability should be accompanied by finance. The participant also said that to involve nurses in research effectively, a beginner must collaborate with an expert in healthcare research development. Participants discussed their experiences with ways of actively engaging individuals in Nigerian society. Financially beneficial and societally honourable activities had positive outcomes. As a result, several participants agreed that utilising research papers as a criterion for employment and promotion would increase research value.

Some have also proposed raising knowledge about research and its advantages, developing research competence among nurses to prepare them for research-related activities and offering incentives to those currently involved in research. According to most participants, supporting interprofessional concord and educational progress for nurses enhances political participation and capacity building. Participants agreed that initiatives should raise awareness about the importance of interprofessional collaboration in the healthcare system, where feedback from all stakeholders is solicited and respected. Participants also emphasised the importance of education in instilling competence and self-confidence. Participants also recognised the need for nurses to become active in healthcare system politics. Because politics pervades all elements of the healthcare system, collective lobbying via the government commissioner was suggested. However, another participant said nurses’ ability in healthcare research formation should be increased before implementing this option to avoid ‘flaws’ that reveal a lack of understanding of the subject. The following are actual quotes that back this up:

‘If you want nurses to be active in research, you should start with their curriculum and work your way up.’ (Participant 3, 45 years old, female)

‘The formal classroom education I get is equivalent to allowing me to pass my exams and go, but if we undertake some in-house training or capacity development, that will pique people’s interest in research.’ (Participant 4, 44 years old, female)

‘“Nurse research awards” would encourage nurses to engage in research activities.’ (Participant 13, 44 years old, female)

‘This will effectively train nurses and sensitise them to healthcare research development.’ (Participant 9, 45 years old, female)

‘The only way to include nurses in research is to have a research workshop followed by research funding.’ (Participant 6, 50 years old, female)

‘If research becomes a requirement for promotion or employment, people will prepare themselves independently by increasing their research abilities and respecting research courses or workshops.’ (Participant 15, 55 years old, female)

Many healthcare research issues must be handled, but a lack of adequate solutions makes them challenging. Healthcare research can solve some of the most pressing challenges confronting the healthcare industry. The application of healthcare research in resolving healthcare issues stems from the fact that the healthcare model differs from traditional business models (Whitehead, Petticrew & Graham 2020). For example, healthcare research may assist the system in resolving cost-control challenges by investing in research to determine cost-control strategies most suitable for the healthcare system. Aside from that, healthcare research may help with revenue definition difficulties. Revenue definition in the healthcare management system may be accomplished by investigating the most potentially lucrative items and services and how to provide them to the market (Hunter 2019). Nurses use several decision-making criteria and methods, recognising experienced nurses as crucial resources. However, incorporating evidence into nursing practice remains challenging for nurses (Nibbelink & Brewer 2018). According to Nibbelink and Brewer (2018), naturalistic decision-making may apply to decision-making nursing research. Nurse experience, the culture of the nurse practice environment, education, nurse comprehension of patient status, situation awareness and autonomy are currently identified as influencing decision-making factors in nursing research. In addition, experienced nurses contribute a diverse set of prior patient contacts to their profession, affecting their intuitive, unconscious processes and aiding decision-making (Nibbelink & Brewer 2018).

## Discussion

The results of this study reveal that research expertise and engagement vary among units and positions and are reliant on identifying research as an essential element of practice, as well as support in the form of time and access to resources. Some participants believe strongly that research is vital, but there is also some conflict linked with the lack of support provided to nurses to conduct research as part of their profession. The previous study on nurses’ opinions of research has shown that research is not viewed as a nursing job, secondary to patient care, and that becoming engaged in research is considered a road away from bedside nursing (Scala et al. 2019). Lack of expertise, time and support are all mentioned as recurring hurdles to nurses doing research (Scala, Price & Day 2016). In keeping with past research, our respondents have a recurring belief that there is insufficient time to do research and a need to prioritise practice over research (Caldwell et al. 2017). The study results are consistent with prior research undertaken with nurses and other healthcare workers (Caldwell et al. 2017). Nurses in the study said they are more inclined to participate in research if they believe it will enhance patient outcomes and if they are included as significant contributors, supported and acknowledged for their work (Caldwell et al. 2017). Without these essential components, research is perceived as an imposition, as too complex and time-demanding. This transition is accompanied by a rise in nurses pursuing higher-level research degrees. As a result, research is often seen as secondary and adversarial to direct patient care. As a result, nurses and midwives are either discouraged from doing research or conducting research on their own time.

## Conclusion

Participants quickly described implementation research projects in which they had engaged or seen considerable effect. The study’s results show that research development allows nurses to participate in research that is important to them and their goals. Despite participants’ varying perspectives on the value and relevance of research, there are a considerable number of nurses working in a variety of clinical settings who are well-positioned and wish to get more active in research. The study’s results will guide policymakers to detect and capitalise on nurses’ growing interest in and readiness for research development.

### Limitations

This study examined nurses’ experiences in participation in research development in Nigeria. The study’s shortcoming was that it did not address other research elements of nursing practice. However, trustworthiness and ethical consideration were applied to reduce the potential influence of the constraint on the study’s richness. In addition, because of the sample size, the study’s findings cannot be extrapolated to larger groups or populations. For example, if the researcher had used a broad sample covering a vast geographical region, the researcher would have received various perspectives on the study issue.
